# Cerebral lesions in hematological malignancies: a case report

**DOI:** 10.1186/s13256-021-03196-4

**Published:** 2021-12-20

**Authors:** Nicoletta D’Ettore, Valentina Scheggi, Brunetto Alterini, Niccolò Marchionni

**Affiliations:** 1 Division of General Cardiology, Cardiothoracovascular Department, Azienda Ospedaliero-Universitaria Careggi, University of Florence, Largo Brambilla 3, 50133 Florence, Italy; 2 Division of Cardiovascular and Perioperative Medicine, Cardiothoracovascular Department, Azienda Ospedaliero-Universitaria Careggi, University of Florence, Florence, Italy

**Keywords:** PML, Rituximab, JC virus, Demyelination, Case report

## Abstract

**Background:**

Progressive multifocal leukoencephalopathy is a rare central nervous system disease, resulting from reactivation of latent John Cunningham virus. Monoclonal antibodies have recently become a relevant risk factor for developing progressive multifocal leukoencephalopathy.

**Case summary:**

We report the case of a 62-year-old Caucasian man who was admitted to our department in June 2020 because of right homonymous hemianopia. Magnetic resonance imaging findings were first interpreted as an intracranial relapsed lymphoma, so brain biopsy was performed, but no neoplastic cell was found. Histological sample only showed a large number of macrophages. The patient came back to our attention because of the worsening of neurological symptoms. A second magnetic resonance imaging showed widespread lesions suggestive of a demyelinating process. John Cunningham virus DNA was detected by polymerase chain reaction assay of the cerebrospinal fluid (over 9 million units/μL). The patient was treated supportively, but the outcome was poor.

**Discussion:**

A multidisciplinary assessment should be performed for differential diagnosis of cerebral lesions in hematologic malignancies. Progressive multifocal leukoencephalopathy should be suspected in cases of subacute neurological symptoms and imaging findings consistent with it, especially if the patient received immunosuppressive or immunomodulatory drugs.

## Background

Progressive multifocal leukoencephalopathy (PML) is a rare central nervous system disease, resulting from reactivation of latent John Cunningham (JC) virus. It is a demyelinating process that may be isolated or, more often, extensively involve both hemispheres. It has a poor clinical outcome; no effective therapy is currently known. It was first observed as a consequence of B-cell lymphoproliferative disorders [1]; starting from the 1980s, acquired immunodeficiency syndrome (AIDS) rapidly became the most common predisposing factor for PML [2]. In the following years, PML was described in patients with autoimmune disorders, so more attention was paid to immunosuppressive therapy [3]. Monoclonal antibodies are considered a relatively new predisposing factor for the development of PML. Cerebral lesions, in addition to cancer and sepsis, can increase the risk of mortality [4, 5].

We report the case of a challenging diagnosis of progressive multifocal leukoencephalopathy in a 62-year-old Caucasian man who received anti-CD20 monoclonal antibody (rituximab) as maintenance therapy for follicular non-Hodgkin lymphoma.

## Case presentation

A 62-year-old Caucasian man was admitted to our department in June 2020. He was diagnosed with follicular lymphoma in 2010 and treated with an R-CHOP regimen (rituximab, cyclophosphamide, doxorubicin, vincristine, prednisolone) for six cycles, achieving complete response; in 2017, because of a disease recurrence, he was treated with six chemotherapy cycles with R-bendamustine, followed by maintenance therapy only with rituximab for 2 years. The last administration was in February 2020. Clinical and radiological follow-up was negative; during the maintenance therapy, the patient had urinary tract recurring infections, gingivitis, and herpes zoster cutaneous reactivation.

He sought medical attention because of progressive vision loss. His pharmacological therapy included atorvastatin, amlodipine, and pantoprazole. He was afebrile; his vital signs were in range. General and neurological physical examination was negative, except for right homonymous hemianopia. Brain computed tomography (CT) showed an uneven cortical and subcortical hypodense lesion in the left posterior temporal and occipital areas, with no contrast enhancement. Magnetic resonance imaging (MRI) confirmed the left parenchymal lesion, composed of two parts: a periventricular one showing inhomogeneous signal (decrease in T1-weighted, increase in T2-weighted sequences); and a more uniform one, involving white matter and characterized by strong T2-weighted and fluid-attenuated inversion recovery (FLAIR) hyperintensity and T1 hypointensity. Restricted diffusion was noted in both components, especially in the white matter, but no gadolinium enhancement was observed. Magnetic resonance (MR) spectroscopy pointed out a reduction of *N*-acetyl aspartate peak, an elevated choline peak, and a double peak of lactic acid.

Total body contrast CT did not document hematological disease activity and confirmed the two previously known lymph nodes, a left axillary and a right mediastinal para-esophageal one, stable in dimensions (16 × 10 and 11 × 6 mm, respectively). Neurological findings were interpreted as a consequence of intracranial relapsed lymphoma. So, after a multidisciplinary assessment, brain biopsy was planned. Histological samples showed a large number of macrophages (CD68^+^, CD14^+^) overshadowing any other cell, except for isolated reactive astrocytes and mild perivascular T-lymphocyte infiltrates. No neoplastic cell was clearly individuated. No microbiological test was performed on brain tissue.

The patient, discharged after brain biopsy, returned to the hospital because of worsening eyesight, mental confusion, and psychomotor slowing. Laboratory investigation on blood was not significant: peripheral blood cell count was normal with 4.61 × 10^9^ white blood cells and mild reduction in hemoglobin level (13.9 g/dL); C-reactive protein was < 5 mg/L. Electrolyte levels, renal function, liver enzyme levels, and coagulation examinations were in range.

**Fig. 1:**
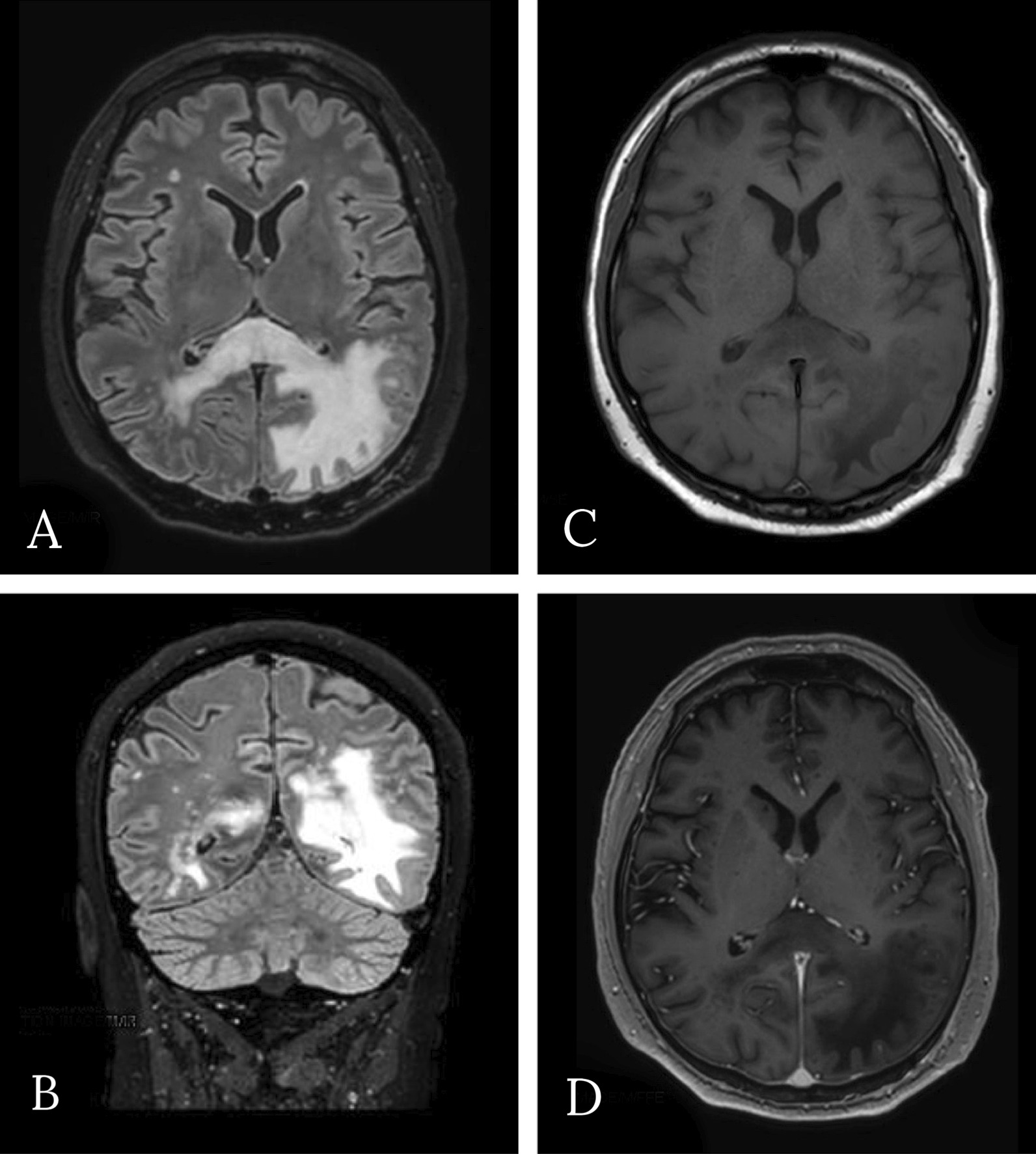
**A, B** Axial and coronal fluid-attenuated inversion recovery; **C** T1-weighted; **D** after administration of gadolinium

**Fig. 2:**
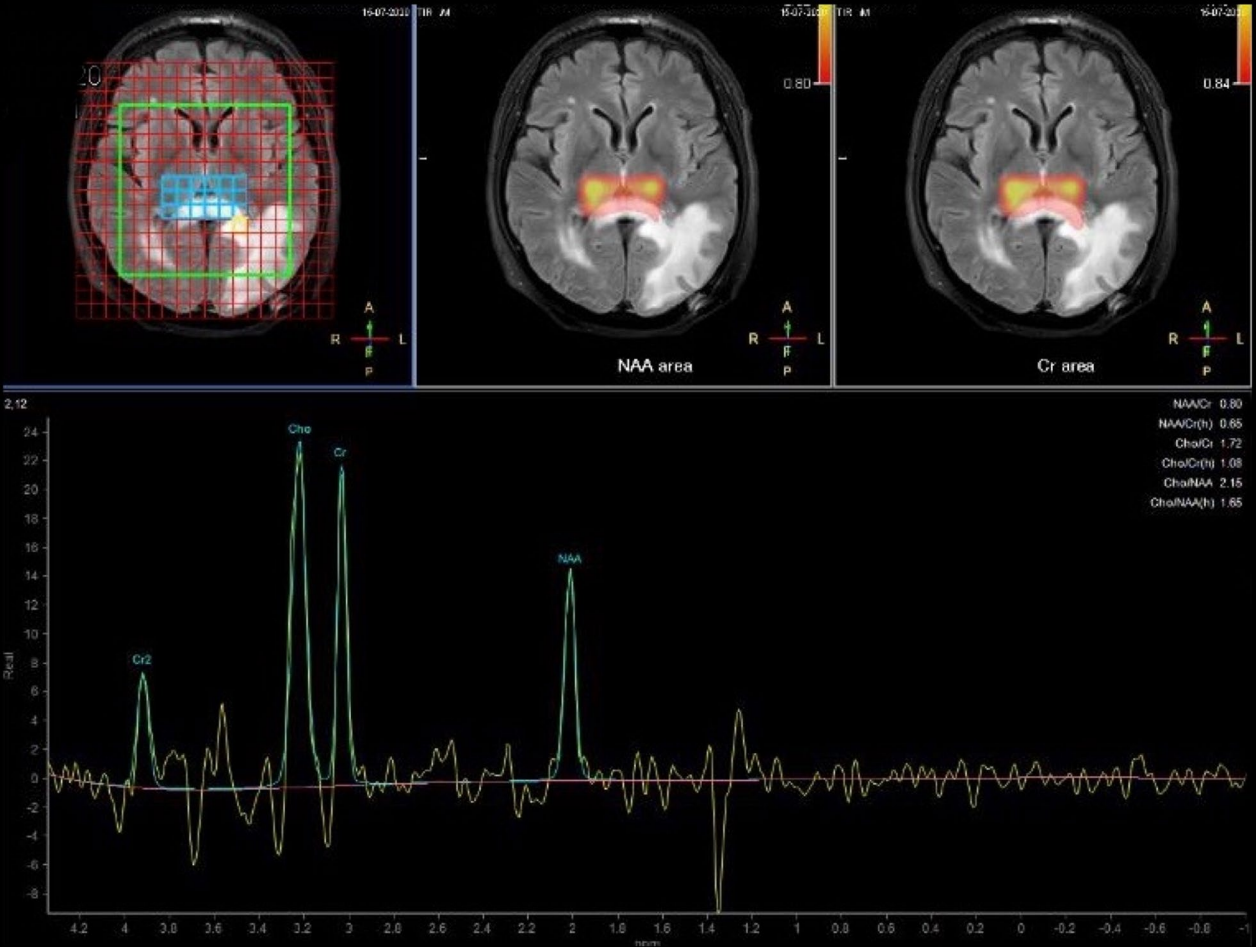
Magnetic resonance spectroscopy

Brain MR was repeated (Figs. 1, 2) and showed, especially on FLAIR/T2-weighted images, the hyperintense left temporal–parietal–occipital and peritrigonal lesion notably increased in dimension and extended from the left to the right hemisphere through the corpus callous. It was also characterized by vasogenic edema with a slight mass effect on cortex gyri. There was no significant contrast enhancement; diffusion-weighted imaging (DWI) showed restricted diffusion around lesion edges. Despite the rapid progression of clinical and imaging findings, the absence of significant contrast enhancement and, above all, the inconclusive response of previous histological report excluded the hypothesis of an expanding neoplastic lesion; MR features were instead considered suggestive for inflammatory demyelinating process, and progressive multifocal leukoencephalopathy was finally suspected. Therefore, a diagnostic lumbar puncture was performed. Cerebrospinal fluid appeared clear and colorless. Chemical–physical analysis showed presence of 10 leukocytes/μL (0.00–5.00) and mild elevation in protein level (albumin 0.350 g/L); glucose level was normal (cerebrospinal-fluid-to-serum ratio 82%). There was an immunoglobulin G (IgG) level of 0.025 g/L and an IgG index (Link index) of 0.66 (< 0.7). Oligoclonal bands were negative. Bacterioscopic and microscopic examination for *Cryptococcus neoformans* was negative; cultural examinations for mycobacteria, *Listeria monocytogenes*, *Borrelia burgdorferi*, and *Toxoplasma* were negative; galactomannan antigen was absent; polymerase chain reaction for Epstein–Barr virus (EBV), cytomegalovirus (CMV), herpes simplex 1 and 2, HHV6, HHV7, HHV8, varicella–zoster, measles, BK virus, adenovirus, enterovirus, Toscana virus, and rubella was negative; virus JC quantitative real-time PCR was positive with detection of numerous viral genomes (9,548,473 units/μL).

CSF serology for aforementioned infectious agents was negative. CFS immunophenotype, despite the low amount of cells after centrifugation, was characterized by 18.0% of lymphocyte, predominantly T, without any B population.

Blood serology for HIV Ag/Ab was negative. Coronavirus disease 2019 (COVID-19) swab test was negative.

He was treated supportively with trazodone and mirtazapine. The outcome was poor: neurological findings continued to worsen, and he progressively became blind, confused, and disorientated. He was finally transferred to a hospice for palliative care (Table 1).

**Table 1:**
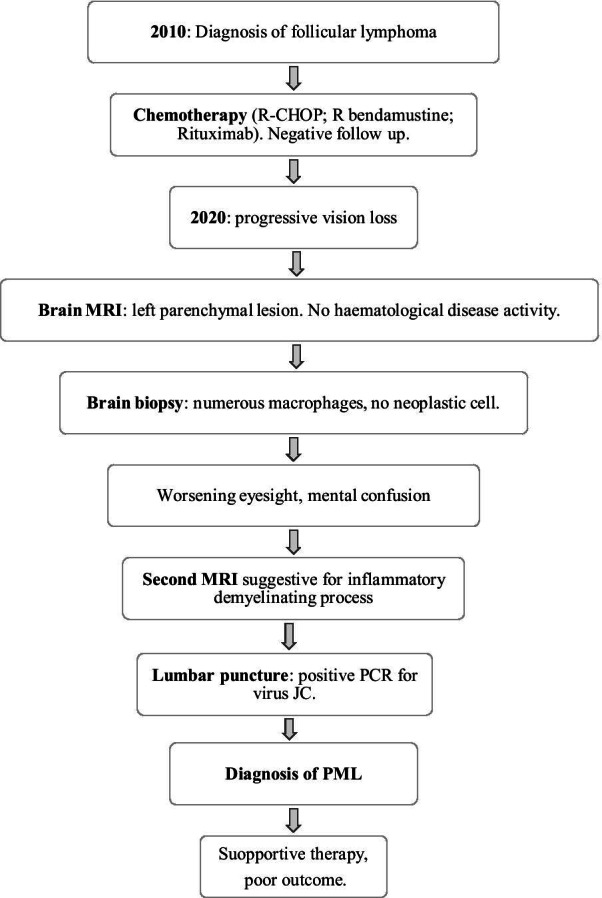
Timetable of the events

## Discussion

Progressive multifocal leukoencephalopathy (PML) is a rare demyelinating disease. It is an opportunistic oligodendrocyte infection, resulting from reactivation of the John Cunningham polyomavirus and generally occurs in immunosuppressed patients. It was firstly described as a consequence of B-cell lymphoproliferative disorders [1], and it often affects allo-HSCT recipients [6]; starting from the 1980s, with the HIV epidemic spread, AIDS rapidly became the most common predisposing factor for PML [2]. In the following years, PML was described in patients with autoimmune disorders, too. Monoclonal antibodies are considered a relatively new predisposing factor to the development of PML [3, 7]: cases were observed among patients treated with natalizumab [8], rituximab, and many other monoclonal antibodies. The incidence of PML with natalizumab and rituximab in HIV-negative patients is 1 in 1000 and 1 in 32,000, respectively; these rates are similar to those in the HIV population (1.3 per 1000) and significantly higher than in the general population (1 per 200,000) [9].

Even if a definitive diagnosis of PML is histopathological, the presence of clinical and imaging manifestations consistent with the diagnosis and not better explained by other disorders, coupled with the demonstration of JC virus by PCR in CSF, is also considered diagnostic [10]. It is important to suspect it in case of cerebral lesions suggestive of a demyelinating disease. PML is usually a multifocal disease. Among patients with hematological malignancies, differential diagnosis is challenging, especially in case of a single area of altered signal. The workup of these patients requires a multidisciplinary approach. Imaging, MRI in particular, plays an important role. MRI findings, including neoplastic lesion (either glioma or lymphoma), ischemia, and infective lesion, can aid in differential diagnosis of PML (Table 2).

**Table 2:** Differential diagnosis

Differential diagnosis	Supportive	Conflicting
Ischemia	Subacute symptoms Brain CT showing single hypodense lesion.	No cerebrovascular territory Expanding lesion
Lymphoma	A single area of altered signal History of hematological malignancy	No hematological disease activity Lymphoma subtype (follicular) Negative histologic sample
Glioma Low grade High grade	A single area of altered signal Imaging (MRI) features Growing speed	No histological feedback Not typical spectroscopy Too fast growth Imaging (MRI) features
Opportunistic infection Bacterial Fungal Viral	Hematological disease Immunomodulatory therapy Relatively frequent Relatively frequent Lumbar puncture positive for JC virus presence	No typical imaging aspect Less common Negative CSF microbiological test No typical imaging aspect Negative CSF microbiological test

Ischemia can be excluded if the lesion does not conform to any cerebrovascular territory and, above all, if it expands significantly on follow-up imaging.

In our case, imaging findings, together with patient history, were extremely suggestive for a central nervous system (CNS) localization of the neoplastic disease. Secondary CNS involvement by systemic lymphoma occurs in approximately 10–15% of patients with Non-Hodgkin lymphoma (NHL), and the incidence depends on histologic subtype: it is more common in aggressive subtypes, such as diffuse large B-cell lymphoma, Burkitt lymphoma, mantle cell lymphoma, and lymphoblastic lymphoma [11]; nevertheless, our patient suffered from follicular non-Hodgkin lymphoma that rarely recurs in the central nervous system, and the absence of any neoplastic cell in histologic samples was unequivocal, eventually.

Low-grade glioma was another interesting alternative because of the imaging findings [intra-axial lesion, hypointense on T1, hyperintense on T2 and FLAIR, without gradient echo (GRE)]. However, spectroscopy reduction of *N*-acetyl aspartate (NAA) peak and choline and lactate peak elevation were less consistent with this hypothesis (high concentration of NAA, low level of choline, and absence of lactate and lipids is more typical) [12]; moreover, it grew too fast. Growing speed was indeed high, similar to a high-grade glioma, but there was no evidence of pathological neovascularization and no necrosis area, hemorrhage, or calcification, and there was no marked mass effect or perilesional edema. Once again, there was no histological confirmation. In the end, metastasis was excluded because of no other detectable neoplastic localization on total-body CT, no distinctive imaging features (such as contrast enhancement, edema, or mass effect), and no histological feedback.

Once neoplastic lesion was excluded, infections became the first assumption, and PML was finally suspected and investigated as described above. Infections of the central nervous system occur in a relevant proportion of immunocompromised patients affected by hematological disorders. Fungal and viral opportunistic infections are common, while bacterial CNS infections are rarely diagnosed, usually in patients with intraventricular devices or after neurosurgical interventions [13]. As mentioned before, PML is caused by JC virus reactivation, and in our case, the pathogenesis was attributed to rituximab therapy.

Rituximab is a chimeric human/murine IgG1 anti-CD20 monoclonal antibody. CD20 receptor is expressed on B lymphocytes, normal and malignant. Rituximab administration is related to a decrease in mature B cells, followed by a pre-B-cell release in peripheral blood. Pre-B cells can be latently infected by the JC virus, after a primary infection that probably occurs in childhood. These lymphocytes can carry the virus in peripheral blood and, through the blood–brain barrier, in the central nervous system [14]. Rituximab is currently approved for the treatment of CD20-positive hematologic malignancies and of systemic and neurologic autoimmune disorders [15]. Fifty-seven PML cases were described among HIV-negative patients treated with rituximab from 1997, date of the first Food and Drug Administration (FDA) approval granted for rituximab, to 31 December 2008; the majority of patients had lymphoproliferative disorders (52) [16]. The pathophysiology of rituximab-associated PML is unclear. In both oncology and autoimmune settings, patients usually have more than one potential risk factor for PML, regardless of rituximab treatment, such as hematological diseases themselves or other cytotoxic immunomodulatory and immunosuppressant agents. Therefore, in this population, PML should be considered a rare but harmful event and may have a multifactorial etiology [17, 18]. Our patient had both lymphoma and monoclonal antibody treatment as risk factors; rituximab may have played an important etiologic role, given the long-term stability of the disease.

PML has a poor prognosis, with high morbidity and mortality. Those who survive can have severe neurological disabilities. Experimental treatment options are emerging [19], but, unfortunately, no effective therapy is currently known, so patients can only be treated supportively. Objective clinical improvement is demonstrated in patients treated with mirtazapine, a serotonin receptor antagonist [20]; the efficacy of this therapy is explained through its antagonism at 5HT_2A_ receptors, the putative cellular receptors for JC virus infection of oligodendrocytes [21].

## Conclusions

As PML is a rare disease and symptoms, as well as imaging features, can be nonspecific, a high level of awareness is necessary for a correct diagnosis. If clinical, laboratory, and imaging findings are consistent with PML, the detection of JC virus by PCR in cerebrospinal fluid is diagnostic. Lumbar puncture is the keystone, whereas a brain biopsy should be performed only when strictly necessary.

Therefore, the assessment of cerebral lesions in a patient with hematological malignancies requires a multidisciplinary approach, and PML should be borne in mind in case of specific risk factor, notably immunosuppressive drugs.

## Data Availability

Not applicable.

## Abbreviations

AIDS: Acquired immunodeficiency syndrome
CSF: Cerebrospinal fluid
CNS: Central nervous system
CMV: Cytomegalovirus
CT: Computed tomography
DWI: Diffusion-weighted imaging
EBV: Epstein–Barr virus
FLAIR: Fluid-attenuated inversion recovery
GRE: Gradient echo
HHV: Human herpes virus
HSCT: Hematopoietic stem cell transplantation
JC: John Cunningham
MR: Magnetic resonance
MRI: Magnetic resonance imaging
NAA: *N*-acetyl aspartate
NHL: Non-Hodgkin lymphoma
PML: Progressive multifocal leukoencephalopathy
PCR: Polymerase chain reaction
5HT_2A_: 5-Hydroxytryptamine
